# Yang-Dan-Tang, Identified from 15 Chinese Herbal Formulae, Inhibits Human Lung Cancer Cell Proliferation via Cell Cycle Arrest

**DOI:** 10.1155/2012/276032

**Published:** 2012-05-30

**Authors:** Tien-Chun Wang, Chih-Nan Fang, Chih-Chien Shen, Hui-Yu Wei, Yui-Ping Weng, Jung-Yaw Lin, Hsiu Mei Hsieh-Li, Chen-Yu Lee

**Affiliations:** ^1^Department of Life Science, National Taiwan Normal University, Taipei 116, Taiwan; ^2^Kuo-Hsing Chinese Clinic, Taipei 110, Taiwan; ^3^Department of Biological Science and Technology, Chung Hwa University of Medical Technology, Tainan 717, Taiwan; ^4^Institute of Biochemistry and Molecular Biology, College of Medicine, National Taiwan University, Taipei 100, Taiwan; ^5^Yu-Sheng Chinese Clinic, Taipei 106, Taiwan

## Abstract

Lung cancer has long been one of the most deadly forms of cancer. The majority of lung cancers are of the non-small-cell lung cancer (NSCLC) type. Here we used the non-small-cell lung carcinoma cell line A549 to screen 15 different traditional Chinese herbal medicine (CHM) formulae to explore the possible mechanisms of alternative medicine in lung cancer therapy. We identified three formulae (Formulae 3, 5, and 14) that substantially decreased the survival of A549 cells but did not affect MRC5 normal lung tissue cells. Formula 14, Yang-Dan-Tang, a modified decoction of Ramulus Cinnamomi Cassiae, was chosen for further characterization. Flow cytometry analysis showed that treatment of Formula 14 induced cell cycle arrest in G1 and G2 phase without causing significant cell death. These results were also confirmed by Western blot analysis, with decreased expression of G1/S and G2/M promoting cell cycle machinery including cyclin D3, cyclin B1, CDK4, and CDK6. This study provides further insight into the possible working mechanism of Yang-Dan-Tang in patients.

## 1. Introduction

Lung cancer, with high incidence and mortality, has long been the most deadly cancer disease. Most lung cancers are carcinomas, and non-small-cell lung carcinoma accounts for more than 80% of these [1]. Although there are many therapeutic approaches toward cancer, none of the treatments can yet substantially cure cancer patients. The development of new drugs and therapies is therefore still of great urgency.

Chinese herbal medicine (CHM) has been used to treat lung cancers for centuries in East Asia. CHM is the most important therapeutic modality of traditional Chinese medicine (TCM). There are more than 130 CHMs that have been reported to have anti-lung-cancer ability [2] and about 40 CHMs [3] and 30 formulae have been frequently used for lung cancer patients [4]. CHMs are usually applied to patients in a form of formula, and each formula may contain more than three herbs. Generally there are one or two major ingredients toward the disease therapy and other ingredients were used to adjust the yin yang extent of the formula according to the patient's status [5, 6]. Some of CHMs are also used in patients under chemotherapies or radiotherapies to reinforce patients' immune activity and metabolism for improving overall prognosis [7, 8]. Although the use of CHMs as therapeutic approaches is very common in East Asia, the specific mechanisms of how CHMs affect the disease still remain largely unknown.

Many studies have used A549 NSCLC lung cancer cells to evaluate the effects of herbs and herb constituents on lung cancers. Plumbagin, phenylbutenoid, and parthenolide were demonstrated to inhibit proliferation of A549 cells [9–11], cantharidin, curcumin, and gambogic acid were reported to induce apoptosis of A549 cells [12–14], and ginsenoside was suggested to have antimetastatic potential toward A549 cells [15]. However, most studies focused on the effects of one single herb toward cancer cells, but not the prescribed multiple herb formula which patients normally take. In this study, we used A549 cells as an NSCLC model to test 15 different CHM formulae which are considered by TCM physicians to have some potential in treating cancers (Table 1).

## 2. Materials and Methods

### 2.1. Cell Culture and Herbal Formula Preparation

The NSCLC cell lines A549 (carcinoma), NCI-H460 (H460, large cell carcinoma), and NCI-H520 (H520, squamous cell carcinoma) and the normal cell line MRC5 were purchased from Bioresource Collection and Research Center (BCRC, Taiwan) and cultured with F12K (for A549), RPMI1640 (for both H460 and H520), and MEM (for MRC5) medium containing 10% FBS, respectively, in a 37°C, 5% CO_2_ incubator. MRC5 cells are normal fetal lung fibroblast cells and used as noncancerous cells in many in vitro lung cancer cytotoxicity studies [16–18]. The 15 different CHM formulae (Table 1) were used to prepare the herbal decoctions as normally prescribed by a CHM doctor for patients. The herbs were boiled in 1.2 L of water for 1 hr to generate the final decoction (around 450 mL), and aliquots were stored at 4°C. To prevent instability and unequal recovery of different ingredients of each formula, concentration under vacuum evaporator was not conducted for all these 15 decoctions. Each formula was applied to cell cultures after decoction debris was removed by passing the liquid through a 0.22 *μ*m membrane filter. 

### 2.2. Cell Proliferation Assay

Three different concentrations of the 15 formula decoctions, 1%, 2%, and 10% (v/v), were applied to A549 cells to determine the IC_50_ of each formula. The maximum 10% decoction was used to avoid dilution of the medium by a large volume of decoction. A549 cells were seeded at 1 × 10^4^ cells per well in a 24-well plate to ensure cells grow logarithmically during total treatment period. Different decoctions were added to the cells 24 hr after seeding, and cell growth was determined by MTT assays after cells were treated for 72 hr.

To test the nontarget effects, IC_50_ of the eight formulae toward A549 (F3: 4%, F4: 8.4%, F5: 2.2%, F6: 9%, F8: 0.4%, F9: 4.2%, F14: 7%, F15: 4%) were applied to A549 and MRC5 cells at the same time. Cell growth was examined by MTT assay after a 72 hr treatment. For time course studies, 7% (v/v) of Formula 14 was applied to A549 cells and MTT assay was performed with these cells after treatment for 0, 24, 48, and 72 hr, respectively. For H460 and H520 cell lines, different amounts of Formula 14 (2–10%) were applied for MTT assay.

### 2.3. Apoptosis Analysis

Characterization of the apoptosis status of A549 cells after Formula 14 (7%) treatment was performed with annexin-V-FLUOS Staining Kit (Roche, Mannheim, Germany) according to the protocol provided by manufacturer. Briefly, 1 × 10^5^ A549 cells were seeded on one 6 cm plate. After treatment for 72 hr, cells were harvested and resuspended with prediluted staining buffer (Annexin-V-Fluos labeling reagent, propidium iodide, and incubation buffer 1 : 1 : 50, resp., 100 *μ*L for 1 × 10^6^ cells). After incubation for 15 min in the dark and sieving through 35 *μ*m nylon mesh, cells were analyzed using FACSCalibur (BD Biosciences, San Jose, CA, USA).

### 2.4. Colony-Forming Assay

The colony-forming assay was modified from previous studies [19, 20]. In brief, A549 and H460 cells treated with Formula 14 (7% and 4.3%, resp.) or vehicle for 72 hr were harvested and seeded onto a 6-cm dish (500 cells) and cultured in a 37°C, 5% CO_2_ incubator for 14 days. The dishes were then removed from the incubator and the medium was discarded. After washing with PBS, the cells were fixed with cold methanol for 10 min. The fixed cells were then stained with crystal violet (Sigma, St. Louis, MO, USA) for 3 min at room temperature. The number of cell colonies in the central 12.25 cm^2^ of the dishes was counted to represent the colony-forming ability of the cells.

### 2.5. Soft Agar Assay

Bacto agar (1.8%) and 2 × F12K/DMEM were mixed (1 : 1) and used to coat the base of a 6-well plate to form a 0.9% agar base. A549 cells treated with Formula 14 (7%) or vehicle for 72 hr were harvested and 1000 cells were mixed with 1.3% methylcellulose and 2 × F12K/DMEM (1 : 1) and seeded onto the 0.9% agar plate. The cells were then incubated at 37°C, 5% CO_2_ in an incubator for one month. Cell colonies in the central 6.5 cm^2^ of the plates were directly counted under microscope to represent the colony-forming ability of cells on soft agar.

### 2.6. Cell Cycle Analysis

A549 cells were seeded at a density of 1 × 10^5^ cells on 6 cm plates, and Formula 14 (7%) was applied to the cells after culture for 24 hr. Cells were incubated for different time periods then fixed with 70% EtOH and stained with propidium iodide for 30 min at 37°C in the dark with frequent shaking. Cells were sieved through a 35 *μ*m nylon mesh and immediately analyzed using FACSCalibur. The percentages of cells at different cell cycle phases were calculated with ModFit software (Verity, Software House, Topsham, ME, USA).

### 2.7. Ki67 Analysis

After treatment with Formula 14 (7%) for 72 hr, cells were fixed and stained with Ki67 antibody and PI using the PE Mouse Anti-Human Ki-67 Set (BD Pharmingen) according to the manufacturer's instruction, with slight modification. Nonspecific PE-conjugated isotype antibody was used as control in this analysis. Both nonstained and single-stained groups were included in the fluorescence compensation. All samples were filtered through 35 *μ*m nylon mesh before entering the flow cytometer (LSR II, BD Biosciences). The data were analyzed with FlowJo software (TreeStar, Ashland, OR, USA).

### 2.8. Western Blot Analysis

Molecules involved in cell cycle regulation were examined using Western blot analyses. A549, H460, and H520 cells were seeded at 2 × 10^5^, 7 × 10^4^, and 5 × 10^5^ cells, respectively, on 10 cm plates and harvested after treatment for 72 hr. Cells were lysed with RIPA buffer (100 mM Tris pH 7.5, 50 mM NaCl, 5 mM EDTA pH 8.0, 1% SDS, 1% sodium deoxycholate, 1% NP40). Ten*μ*g protein of each sample was electrophoresed by SDS-PAGE and then transferred onto Hybond-C extra membrane (Amersham, GE Healthcare, UK). The membrane was blocked with 5% nonfat dry milk powder in Tris-buffered saline with 0.1% Tween-20 for 1 hr at room temperature. After blocking, the membrane was incubated with the primary antibody [Cyclin D3, 1 : 1000; CDK4, 1 : 1000; CDK6, 1 : 1000; cdc2, 1 : 1000; pcdc2, 1 : 1000 and pRb,1 : 1000 (Cell Signaling, Danvers, MA, USA); cyclin B1, 1 : 1000 (Upstate, Lake Placid, NY, USA)]. The corresponding HRP-conjugated secondary antibody was applied to the membrane for 1 hr to highlight the specific signal. HRP substrate (Millipore, MA. USA) was added onto blot membrane to show the hybridization pattern, and images were acquired with imaging system LAS-3000 (Fujifilm, Japan). Blot images were quantified with Multi-Gauge software (Fujifilm).

### 2.9. Statistical Analysis

Data are presented as mean ± SE. Student's *t*-test was performed with SPSS software to evaluate the significance of differences between group means. A *P-*value of less than 0.05 was considered to be significant.

## 3. Results

### 3.1. Cytotoxicity Analysis of 15 TCM Formulae

MTT assays were performed with the NSCLC cell line A549 after treatment with different formulae for 72 hr. Of the 15 formulae, Formulae 3, 4, 5, 6, 8, 9, 14, and 15, substantially reduced the growth rate of A549 cells over 50% at the highest 10% (v/v) dosage (Table 2). The IC_50_ of the 8 formulae were calculated using the interpolation method and are listed in Table 2. Among these 8 formulae, Radix Scutellariae Baicalensis is one of the dominant ingredients in 7 of them, except for Formula 5. Radix Scutellariae Baicalensis is also an ingredient of Formula 7 although 10% (v/v) reduced cell viability to 61%. Whether Radix Scutellariae Baicalensis is the major component that decreases the growth of cells needs further investigation.

**Figure 1:**
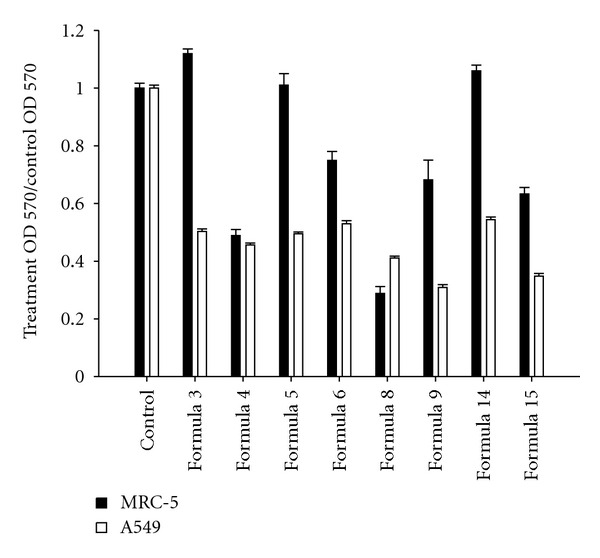
Effect of 8 CHM formulae on the cytotoxicity of A549 cells. Survival rate of A549 and MRC5 cells following treatment at the IC_50_ with the 8 chosen formulae for 72 hr was analyzed by MTT assay. Data were normalized to control groups. Formulae 3, 5, and 14 decreased the survival rate of A549 cells around 50% without affecting that of MRC5 cells.

To test whether these 8 active formulae have any significant nontarget effect, the normal lung cell line MRC5 was included in the cytotoxicity screening. The eight formulae were used to treat A549 and MRC5 cell lines for 72 hr at their IC_50_ toward A549. We found that Formulae 3, 5, and 14 reduced the growth rate of A549 cells around 50% without significantly affecting the viability of MRC5 cells (Figure 1). In contrast, the other 5 formulae (4, 6, 8, 9, and 15) showed significant nontarget effects, suppressing the growth rate of MRC5 about 20–70% (Figure 1). Among the three A549-specific formulae, Formula 14, Yang-Dan-Tang, a modified decoction of Ramulus Cinnamomi Cassiae, was chosen for further study due to its traditional CHM use as therapy for lung disease.

### 3.2. CHM Formula 14 Reduced A549 Cells Proliferation without Inducing Massive Apoptosis

**Figure 2:**
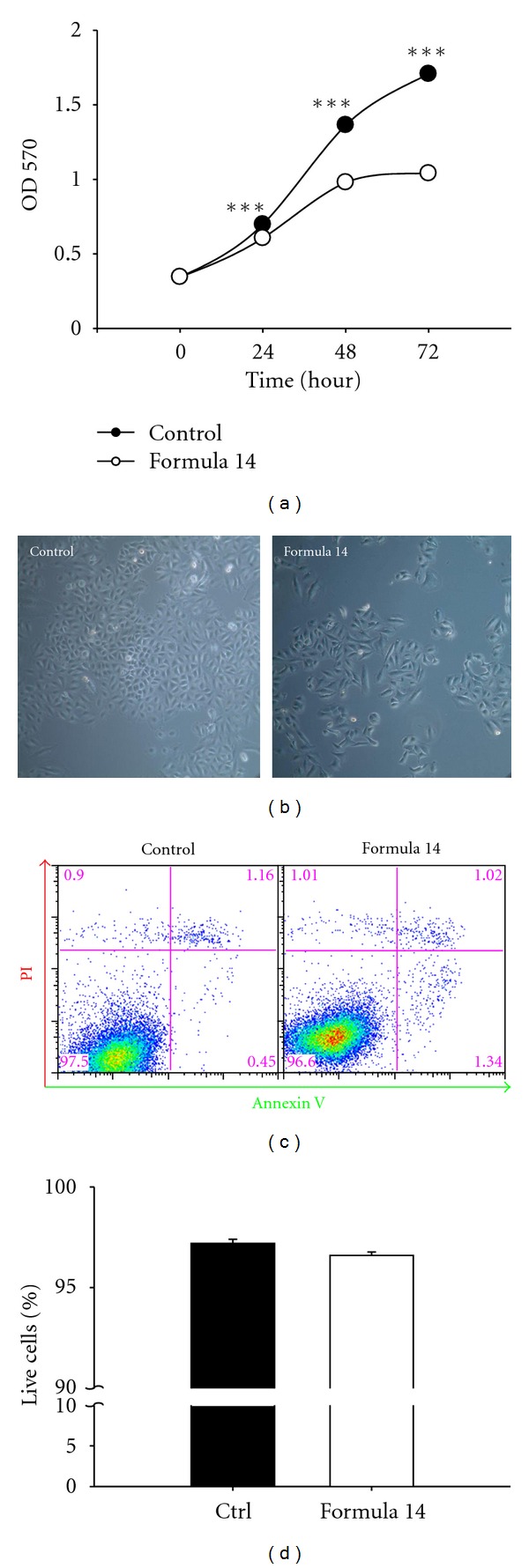
CHM Formula 14 decreased A549 cell survival without apparent apoptosis. A time course MTT assay was used to investigate the response of A549 cells to treatment with Formula 14. (a) Formula 14 treatment significantly inhibited the proliferation of A549 cells at all the time points studied. (b) Bright field images of A549 cells after 72 hr treatment with vehicle or Formula 14. The cell density of A549 cells after treatment with Formula 14 was much lower than the control group. (c) Representative data of annexin V and PI double staining show the apoptosis/necrosis status of A549 cells after 72 hr treatment with Formula 14. (d) Percentages of the double negative live cells (bottom left square) were quantified to compare the healthy population. Formula 14 has no effect on the live cell percentage. Student's *t-*test:,***P < 0.001.

To further elucidate the growth inhibiting mechanisms of Formula 14, the time course of the response of A549 cells to Formula 14 treatment was examined. Formula 14 could significantly decreased the A549 cell numbers after 24 hr treatment (Figure 2(a)), suggesting that Formula 14 could either actually kill the cells or inhibit the proliferation of the cells. The cell density of the Formula 14 treated group was much lower than that of control group; however, few dead cells could be seen under microscope observation (Figure 2(b)).

To further clarify the two possibilities, Annexin V and propidium iodide double staining was performed to see if cell death occurred after treatment. No significant difference was identified for the healthy populations between Formula 14-treated and control groups (Figures 2(c) and 2(d)). In addition, little cell death (early apoptosis, late apoptosis, or necrosis) was identified in both the Formula 14-treated and control groups (Figure 2(c)). These results suggest that Formula 14 treatment reduction of the cell number might be through inhibition of proliferation.

### 3.3. Effects of CHM Formula 14 on the Decrease of A549 Cell Colony Formation

**Figure 3:**
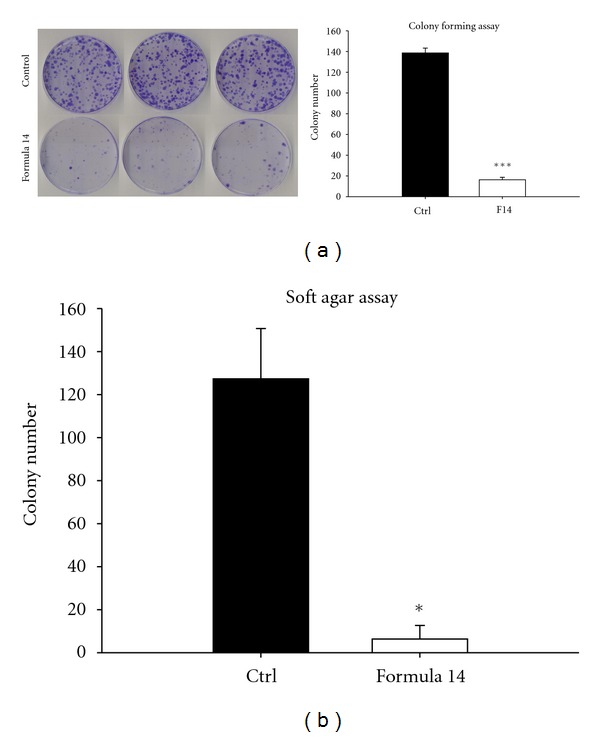
CHM Formula 14 decreased the colony formation activity of A549 cells. (a) Cell colonies were stained with crystal violet and scanned to generate images. Colony numbers of those cells treated with Formula 14 (lower panel) were significantly decreased. (b) Anchorage-independent growth ability. Colony numbers were significantly decreased by treatment with Formula 14 for 72 hr. Student's *t*-test: *, ***P < 0.05 and 0.001, respectively.

We also conducted colony forming-assay to determine whether Formula 14 could affect the tumorigenesis ability of A549 cells, and the results showed that the number of colonies at 14-day culture was significantly reduced in cells pretreated with Formula 14 compared to that of the control group (Figures 3(a) and 3(b)). In addition, the formation of colonies on soft agar after one month was also dramatically decreased in the Formula 14-treated cells (Figure 3(c)). These two different assays revealed that Formula 14 could reduce tumorigenesis in A549 cells.

### 3.4. CHM Formula 14 Induced A549 Cell Cycle Arrest at G1 and G2 Phases

**Figure 4:**
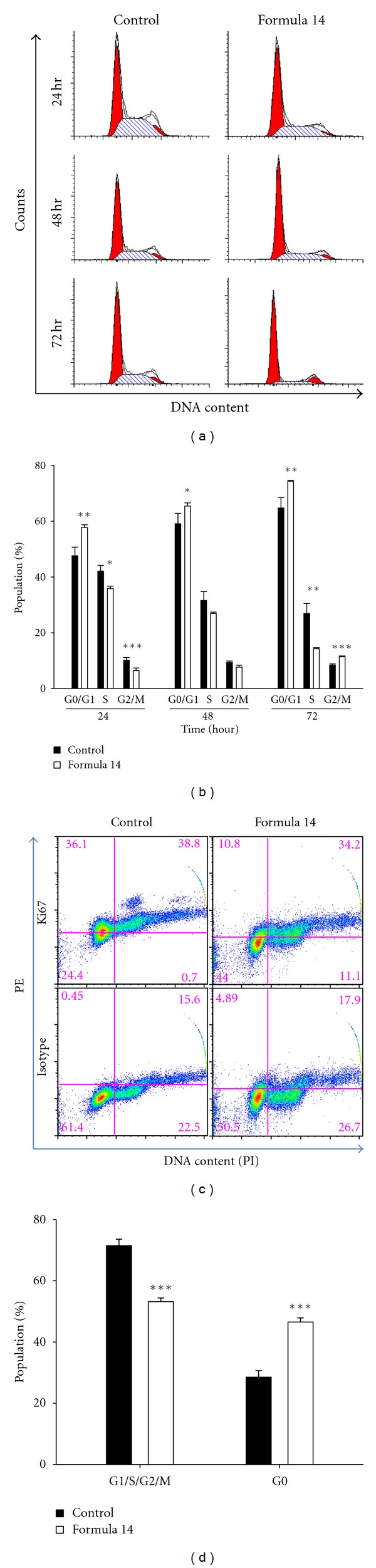
CHM Formula 14 led to A549 cells arrested in G0/G1 and G2 phase. Cell cycle analysis of A549 cells after treatment with Formula 14. (a) Representative data of cell cycle distribution profile under Formula 14 treatment. Treatment with Formula 14 led to significant cell cycle arrest in G1 and G2 phase. (b) Quantified result of cells at different phases from (a). (c) Representative data of ki67 analysis of A549 cells after a 72 hr treatment of Formula 14. (d) Fraction of cells in G0 and other phases. Formula 14 significantly increased the G0 cell population after 72 hr treatment. Student's *t*-test: *, **, ***P < 0.05, 0.01, and 0.001, respectively.

We then tested whether Formula 14 affected cell cycle progression, and the results showed that the number of cells in the proliferative S phase was significantly decreased whereas cell populations in G1 and G2 phases were significantly increased after 24 and 72 hr treatment, respectively (Figures 4(a) and 4(b)). However, there was no cell population identified in the subG1 phase (Figure 4(a)). These data indicate that CHM Formula 14 treatment did not induce cell apoptosis but rather reduced the proliferation rate of A549 cells. In addition, a Ki67 analysis showed there were more cells in the quiescent G0 phase after treatment with Formula 14, compared to that of the control group (Figures 4(c) and 4(d)). In conclusion, CHM Formula 14 retarded cell cycle progression significantly.

### 3.5. Effect of CHM Formula 14 on the Expression Level of Cell Cycle Related Genes

**Figure 5:**
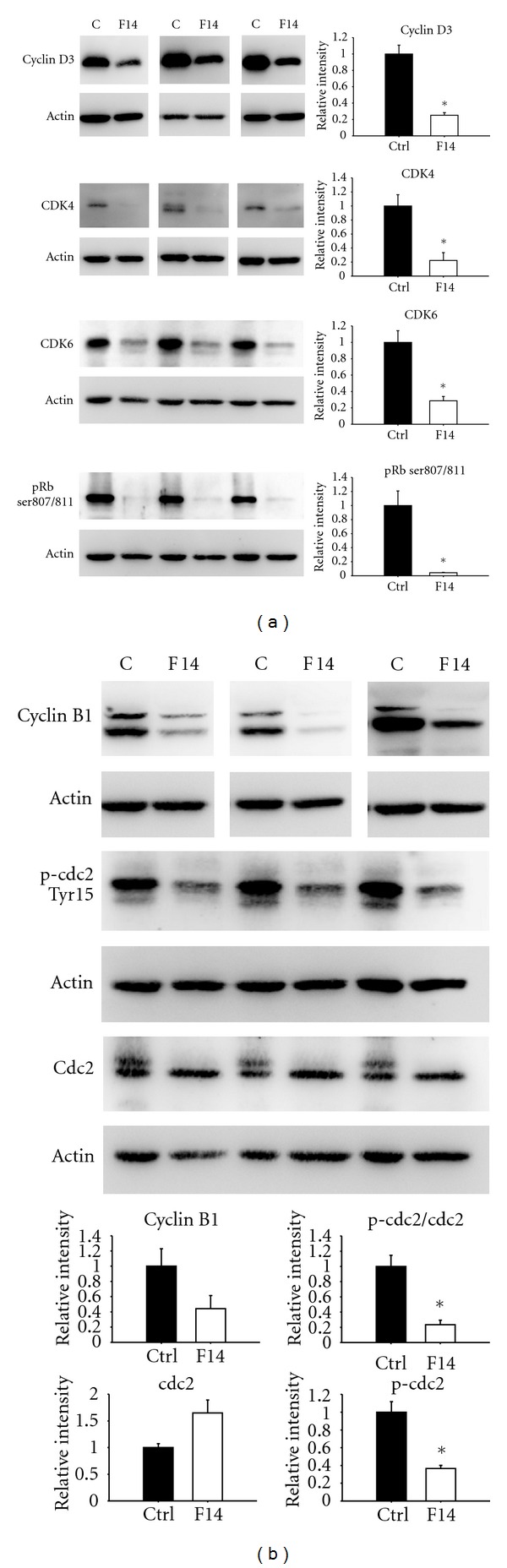
Western blots of the G1/S and G2/M regulatory proteins in A549 cells after 72 hr treatment of Formula 14. (a) Examination of A549 G1/S regulatory protein levels after treatment with Formula 14 for 72 hr. The expression levels of cyclin D3, CDK4, and CDK6 and the phosphorylation of Rb were significantly decreased after treatment with Formula 14. (b) Western blots of cyclin B1, phosphorylated cdc2, and total cdc2. The expression levels of cyclin B1 and the phosphorylation of cdc2 were decreased, while the expression of cdc2 was increased after treatment with Formula 14. Results of 3 independent experiments were shown at the same time to show the consistent results. Student's *t*-test: *P < 0.05. (*n* = 3).

We then examined the expression of cell cycle regulatory proteins to uncover more molecular clues to the effects of Formula 14. Western blot analyses showed that expression of G1/S transition promoting proteins, including cyclin D3, D-type-cyclin-dependent kinase CDK4 and CDK6, and phosphorylated retinoblastoma protein (pRb), the substrate of cyclin D/CDK4/6 complex, was decreased by Formula 14 (Figure 5(a)). The reduced levels of these molecules could result in G1 phase arrest, which is in agreement with the result of the previous cell cycle analysis. In addition to the G1 phase arrest, the G2 phase cell population was also increased in A549 cells after treatment with Formula 14 for 72 hr (Figure 3(a)). We thus further checked the G2/M transition regulating proteins, cyclin B1 and cdc2 (cdk1). Treatment with Formula 14 decreased the expression of both cyclin B1 and phosphorylated cdc2 (Tyr15-p-cdc2) and increased the total amount of all forms of cdc2 (Figure 5(b)). The downregulation of cyclin B1 is in accordance with the G2 arrest effect of Formula 14, while the decreased ratio of Tyr15-p-cdc2/total cdc2 may result from the mixed composition of Formula 14.

### 3.6. Formula 14 Has Similar Effects on Other NSCLC Cell Lines

**Figure 6:**
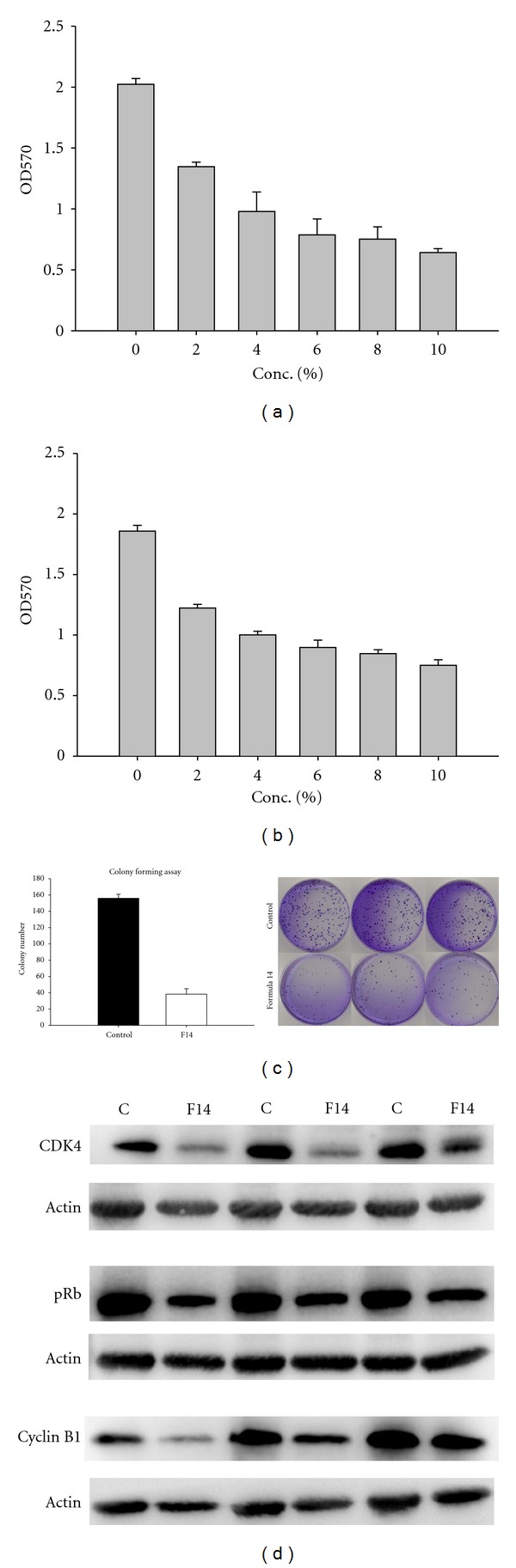
The effect of Formula 14 on NSCLC cell lines H460 and H520. Survival rate of H460 (a) and H520 (b) cells following treatment with Formulae14 for 72 hr was analyzed by MTT assay. (c) Formula 14 decreased the colony formation activity of H460 cells. Cell colonies were stained with crystal violet and scanned to generate images. Colony numbers of those cells treated with Formula 14 (lower panel) were significantly decreased. (d) Western blots of the cell cycle regulatory proteins in H520 cells after 72 hr treatment of Formula 14. Results of 3 independent experiments were shown at the same time to show the consistent results.

To examine whether Formula 14 has similar cytotoxicity toward other NSCLC cells, we further applied Formula on H460 (large cell carcinoma) and H520 (squamous cell carcinoma) cell lines. Our results showed that Formula 14 also reduced the proliferation of both H460 and H520 cells (Figures 6(a) and 6(b)). The IC_50_ for H460 and H520 are 4.5% and 3.8%, respectively. In addition, Formula 14 also reduced the colony-forming activity of H460 cell line. The number of colonies at 14-day H460 culture was significantly reduced in cells pretreated with Formula 14 compared to that of the control group (Figure 6(c)). The Western blot analyses showed that Formula 14 decreased the expression levels of cell cycle regulatory proteins, CDK4, pRb, and cyclin B1 of H520 cell line (Figure 6(d)). These data showed that Formula 14 has a general effect on different NSCLC cell lines.

## 4. Discussion

In this study, we used the A549 cell line as an NSCLC model to screen 15 different CHM formulae (Table 1). Some of the 15 formulae are frequently prescribed to cancer patients while the others were considered by TCM physicians to have some potential in treating cancers. Formulae 3, 5 and 14 were identified to substantially reduce the growth of A549 cells but not MRC5 normal lung tissue cells. Formula 14 was used for further investigation in light of its CHM traditional use in lung disease therapy.

Our study reveals that Formula 14 decreased the in vitro tumorigenesis of A549 cells. The A549 lung cancer cell line has been used widely for decades and has stable tumorigenicity. Here, however, cells treated with Formula 14 showed significantly reduced colony formation in both media and soft agar over long culture periods (14–30 days) compared to the control cells. Human lung tumor xenografts in immunosuppressed mice will be conducted in future to explore the in vivo antitumorigenic effects of Formula 14 treatment.

The growth of A549 cells was significantly reduced by Formula 14 treatment, which could be a result of either inhibiting proliferation or increasing cell death. We suggest that the cell growth reduction by Formula 14-treatment must be attributed to a specific inhibition of cell proliferation rather than induction of apoptosis. The annexin V and PI double staining experiments showed that Formula 14 treatment did not influence the A549 cell distribution in normal (both annexin V and PI negative), early apoptotic (annexin V positive and PI negative), and late apoptotic or necrotic (both annexin V and PI positive) populations. Fewer than 2% of cells were distributed in the early or late apoptosis populations. In addition, the A549 cell cycle profile obtained from the PI staining revealed there was no subG1 (apoptotic) cell population. All of these observations suggest that Formula 14 did not induce apoptosis in A549 cells. Indeed, the cell cycle analyses showed that both G1 and G2 phase arrest were induced in A549 cells by Formula 14. These data together explain why Formula 14 suppressed the cell growth without causing the cell death. We also noticed significant G1 arrest in cells treated with Formula 14 for only 24 hr, while G2 arrest was not evident until 72 hr had elapsed.

We also examined the expression levels of regulators involved in the cell cycle. Substantial decline of the G1/S regulatory proteins (cyclin D3, CDK4, and CDK6) was identified by Western blot analysis, which is in accordance with the G1 arrest results obtained from cell cycle analysis. We found that the phosphorylation of Rb (Ser807/811) was also downregulated after treatment. Rb is a tumor suppressor protein that regulates cell proliferation by controlling progression through the G1 restriction point of the cell cycle. Phosphorylation by cyclin D-CDK4/6 and cyclin E-CDK2 causes Rb inactivation, thus allowing cell cycle progression [21–23]. Our result indicates that levels of these critical regulators involved in G1/S promotion were severely reduced by Formula 14.

However, Formula 14 seemed to have a more complicated effect toward G2/M cell cycle regulation. Formula 14 treatment decreased not only the expression of cyclin B1 but also the Tyr-15 phosphorylated (pY15) form of cdc2. Cell cycle G2/M progression is regulated by the cdc2-cyclin B1 kinase complex and is maintained in an inactive form by phosphorylation at Thr14/Tyr15 of cdc2 [24]. Entry into the M-phase of the cell cycle is regulated by activation of cdc2 kinase through cyclin B1 binding and dephosphorylation of cdc2 at Thr14/Tyr15 [25, 26]. Although the level of Tyr15-p-cdc2 was reduced by Formula 14, which would tend to promote the G2/M progression, the severe reduction of cyclin B1 would tend to decrease the formation of cyclin B1-cdc2 complex. We suggest that the accumulation of cells in G2/M after Formula 14 treatment for 72 hr might be caused by the profound reduction of cyclin B1. We suspect that different ingredients in Formula 14 have different targets toward the cell cycle, which will need further investigation.

To further investigate whether Formula 14 has a general effect toward NSCLC cell lines, we examine the viability of another two NSCLC cell lines, H460 and H520, after treatment with Formula 14. The study showed that IC_50_ of these two cell lines were smaller than that of A549 cells, which suggest Formula 14 could inhibit the growth of H460 and H520 effectively. Results of colony-forming and Western blot analyses further provide the evidence that Formula 14 could suppress the tumorigenicity and cell cycle progression activities of H460 and H520 cells. Based on these observations, we conclude that Formula 14 has similar effect on NSCLC cell lines.

Formula 14 is the standard formulation of Gui-Zhi-Tang with the addition of *Scutellariae baicalensis *to create another formula known as Yang-Dan-Tang. It was recently reported that the crude ethanolic extracts of *Scutellariae baicalensis* were selectively toxic to human lung cancer cell lines [27]. Several investigations on constituent herbs of Formula 14 have revealed several pure compounds possessing various biological activities. Isoliquiritigenin isolated from *Glycyrrhizae uralensis* has been shown to induce apoptosis in colorectal cancer cell lines [28]. Baicalein, baicalin, oroxylin A, and wogonin are effective compounds from *Scutellariae baicalensis* and were identified to have antiproliferation and proapoptosis ability [29–32]. Crude extracts of *Fructus Zizyphi Jujubae* and *Rhizoma Zingiberis* were shown to block angiogenesis [33] and enhance paclitaxel sensitivity [34], respectively. Water extract of *Paeoniae Rubrae* was also reported to have apoptotic effect on hepatocellular carcinoma cells [35]. In our study, we found Formula 14 could induce cell cycle arrest, as several previous reports also show; however, we did not observe obvious apoptosis of A549 cells after treatment with the formula. One possible explanation is there might be interaction between different herb constituents of this formula which could lead to complicated effects. Another factor that might affect the herb effects is serum concentration in the growth media. Most studies observed apoptotic events when using a low serum (0~5%) condition [36–38], whereas we used a higher serum (10%) condition in this study, which could provide stronger survival signals or, alternatively, generate a stronger serum binding effect to compromise the herb effects. On the other hand, Formula 14 could not induce apoptosis but arrest cells at G1/G2 phases of cell cycle, suggesting a cytostatic activity for lung tumor. The significance of cytostasis in anticancer drug activity drew more attention as drug development efforts have moved from conventional chemotherapy to target therapy. However, it is suggested that a prolonged arrest in a cell cycle stage other than G0 is intolerable to a cell and must be resolved by either initiating a cell death or escaping from the cell-cycle block [39]. Therefore, it is important to examine the issue whether a continuous long-term administration of Formulas 14 could have tumor-shrinkage effect in the further investigation with xenograft mouse model.

## 5. Conclusion

We identified 3 potential formulae that could substantially decrease the survival of NSCLC cells while not affecting MRC5 normal lung cells. Formula 14, the Yang-Dan-Tang, was chosen for further study for its effect on cell growth suppression. A colony-forming assay demonstrated that Formula 14 has antitumorigenic activity. In addition, flow cytometry showed that Formula 14 induced cell cycle arrest in G1 and G2 phase without causing significant cell death. This result was further confirmed by western blot analysis which showed reduced expression of G1/S and G2/M regulators. Our study provides further insight into the working mechanism of Chinese medicine formulae in patients.

## Figures and Tables

**Table 1:** The composition of 15 herbal formulae.

Formula 1	Angelicae Sinensis 11.25 g, Ligustici Chuanxiong 11.25 g, Paeoniae Rubrae 11.25 g, Rehmanniae Glutinosae 11.25 g, Gummi Olibanum 11.25 g, Myrrha 11.25 g, Atractylodis Rhizoma 11.25 g, Raw Radix Glycyrrhizae Uralensis 11.25 g, Semen Persicae 11.25 g, Flos Carthami Tinctorii 1.875 g

Formula 2	Angelicae Sinensis 11.25 g, Ligustici Chuanxiong 11.25 g, Paeoniae Rubrae 11.25 g, Rehmanniae Glutinosae 11.25 g

Formula 3	Rhizoma Coptidis 11.25 g, Radix Scutellariae Baicalensis 11.25 g, Cortex Phellodendri 11.25 g, Fructus Gardeniae Jasminoidis 11.25 g, Raw Radix Glycyrrhizae Uralensis 18.75 g, Atractylodis Rhizoma 15 g

Formula 4	Radix Gentianae Longdancao 11.25 g, Radix Scutellariae Baicalensis 11.25 g, Fructus Gardeniae Jasminoidis 7.5 g, Angelicae Sinensis 11.25 g, Rehmanniae Glutinosae 11.25 g, Caulis Mutong 11.25 g, Radix Bupleuri 11.25 g, Semen Plantaginis 11.25 g, Atractylodis Rhizoma 15 g, Rhizoma Alismatis Orientalis 15 g, Raw Radix Glycyrrhizae Uralensis 18.75 g

Formula 5	Rhizoma Coptidis 11.25 g, Rhizoma Cimicifugae 11.25 g, Angelicae Sinensis 11.25 g, Rehmanniae Glutinosae 11.25 g, Cortex Moutan Radicis 11.25 g

Formula 6	Fructus Forsythiae Suspensae 11.25 g, Herba Menthae Haplocalycis 11.25 g, Fructus Gardeniae Jasminoidis 11.25 g, Radix Scutellariae Baicalensis 11.25 g, Herba Lophatheri Gracilis 11.25 g, Raw Radix Glycyrrhizae Uralensis 11.25 g, Radix et Rhizoma Rhei 3.75 g, Mirabilitum 3.75 g, Atractylodis Rhizoma 15 g

Formula 7	Pericarpium Citri Reticulatae 15 g, Rhizoma Pinelliae Ternatae 15 g, Sclerotium Poriae Cocos 15 g, Raw Radix Glycyrrhizae Uralensis 15 g, Radix Scutellariae Baicalensis 11.25 g, Fructus Immaturus Citri Aurantii 11.25 g, Caulis Bambusae in Taeniis 3ea, Fructus Zizyphi Jujubae 5ea

Formula 8	Angelicae Sinensis 11.25 g, Ligustici Chuanxiong 11.25 g, Paeoniae Rubrae 11.25 g, Rehmanniae Glutinosae 11.25 g, Radix Glycyrrhizae Uralensis 11.25 g, Radix Scutellariae Baicalensis 11.25 g, Cortex Lycii Radicis 18.75 g, Cortex Moutan Radicis 18.75 g, Atractylodis Rhizoma 15 g

Formula 9	Angelicae Sinensis 11.25 g, Ligustici Chuanxiong 11.25 g, Radix Paeoniae Lactiflorae 11.25 g, Rehmanniae Glutinosae 11.25 g, Fried Atractylodis Macrocephalae 11.25 g, Radix Glycyrrhizae Uralensis 11.25 g, Radix Dipsaci Asperi 11.25 g, Cortex Eucommiae Ulmoidis 11.25 g, Radix Scutellariae Baicalensis 11.25 g, Radix Dioscoreae Oppositae 18.75 g, Folium Artemisiae Argyi 18.75 g

Formula 10	Radix Achyranthis Bidentatae 30 g, Radix Dipsaci Asperi 30 g, Rhizoma Drynariae 30 g, Rubiaakane Nakai 30 g, Raw Radix Glycyrrhizae Uralensis 18.75 g, Atractylodis Rhizoma 15 g

Formula 11	Angelicae Sinensis 30 g, Radix Polygoni Multiflori 30 g, Semen Cuscutae Chinensis 30 g, Semen Astragali Complanati 30 g, Tribulus terrestris Linn 15 g, Atractylodis Rhizoma 15 g

Formula 12	Angelicae Sinensis 11.25 g, Ligustici Chuanxiong 11.25 g, Paeoniae Rubrae 7.5 g, Rehmanniae Glutinosae 11.25 g, Radix Salviae Miltiorrhizae 18.75 g, Atractylodis Rhizoma 18.75 g, Radix Astragali Membranacei 56.25 g

Formula 13	Radix Astragali Membranacei 75 g, Angelicae Sinensis 15 g, Ligustici Chuanxiong 15 g, Paeoniae Rubrae 15 g, Ginkgo Bilobae 15 g

Formula 14	Ramulus Cinnamomi Cassiae 18.75 g, Paeoniae Rubrae 18.75 g, Raw Radix Glycyrrhizae Uralensis 18.75 g, Rhizoma Zingiberis Officinalis Recens 18.75 g, Radix Scutellariae Baicalensis 11.25 g, Fructus Zizyphi Jujubae 5ea

Formula 15	Rhizoma Cimicifugae 7.5 g, Rehmanniae Glutinosae 56.25 g, Paeoniae Rubrae 37.5 g, Cortex Moutan Radicis 37.5 g, Radix Scutellariae Baicalensis 37.5 g

**Table 2:** Cytotoxicity of 15 CHM formulae against A549 cells.

Formula (v/v)	1%	2%	10%	IC_50_ conc.
Formula 1	111%	98%	77%	NA
Formula 2	106%	109%	95%	NA
Formula 3*	67%	60%	32%	4%
Formula 4*	96%	96%	35%	8.4%
Formula 5*	57%	46%	28%	2.2%
Formula 6*	89%	83%	29%	9%
Formula 7	105%	106%	61%	NA
Formula 8*	24%	11%	5%	0.4%
Formula 9*	107%	80%	7%	4.2%
Formula 10	104%	86%	51%	NA
Formula 11	83%	87%	65%	NA
Formula 12	94%	95%	69%	NA
Formula 13	104%	102%	62%	NA
Formula 14*	97%	77%	41%	7%
Formula 15*	76%	70%	12%	4%

*Indicates the proliferation inhibition exceeds 50% under the maximum dose.
